# Case Report: Thoracic duct ligation for left-sided chylothorax after pneumonectomy with contralateral VATS procedure using indocyanine green fluorescence

**DOI:** 10.3389/fsurg.2025.1558519

**Published:** 2025-04-29

**Authors:** Chiara Anna Schiena, Mario Pezzella, Eleonora Faccioli, Alessandro Rebusso, Giovanni Comacchio, Stefano Silvestrin, Michele Battistel, Edoardo Rosellini, Andrea Dell’Amore, Federico Rea, Samuele Nicotra

**Affiliations:** ^1^ Thorac Surgery Unit, Department of Cardiac, Thoracic, Vascular Sciences and Public Health, University of Padua, Padua, Italy; ^2^ Department of Medicine, Institute of Radiology, University Hospital of Padua, Padua, Italy; ^3^ Department of Medicine, Institute of Anesthesia and Intensive Care, University Hospital of Padua, Padua, Italy

**Keywords:** thoracic surgery, pneumonectomy, chylothorax, thoracic duct ligation, ICG fluorescence

## Abstract

**Introduction:**

Chylothorax is a rare but potentially life-threatening condition characterized by the accumulation of lymphatic fluid in the pleural cavity. It is typically managed with conservative treatments such as fasting and/or thoracic duct embolization via lymphography. However, when these approaches fail, surgical intervention, most commonly thoracic duct ligation (TDL), is often necessary. While the advent of video-assisted thoracoscopic surgery (VATS) has enabled minimal invasive approaches for thoracic duct ligation, intraoperative identification of the thoracic duct remains technically challenging.

**Case report:**

We present the case of a 62-year-old man diagnosed with SMARCB1-deficient mediastinal sarcoma who underwent left pneumonectomy and subsequently developed a left-sided chylothorax on postoperative day 16. Initial management with conservative strategy first, including two lymphography procedures with attempted embolization, was unsuccessful. Consequently, we proceeded with thoracic duct ligation via right-sided VATS, employing indocyanine green (ICG) fluorescence to aid in the identification of the thoracic duct. Given the prior left pneumonectomy, a single-lumen endotracheal tube with a bronchial blocker was used to selectively exclude the right lower lobe during the procedure.

**Conclusion:**

This case highlights the use of ICG fluorescence in facilitating the identification and ligation of the thoracic duct in a patient with left-sided chylothorax following left pneumonectomy.

## Introduction

Chylothorax is the accumulation of chyle in the pleural cavity, most commonly resulting from iatrogenic injury to the thoracic duct or its tributaries (1). If not promptly treated, this condition can be fatal in up to 30% of patients (2). In many cases, conservative managements including fasting, fat-free diets, and chest drainage- proves effective. However, when these measures fail, or when chyle output exceeds 1,000 ml/day, more invasive interventions, such as thoracic duct ligation, may become necessary (3). Lymphography plays a valuable role in both diagnosing and, in some cases, resolving chylothorax by localizing lymphatic leaks (2). If conservative approaches are unsuccessful, surgical management is indicated. An emerging intraoperative technique to facilitate visualization of the thoracic duct is the use of indocyanine green (ICG) fluorescence. Under generally anesthesia, the patient is initially placed the supine position, for bilateral injection ICG into the inguinal lymph nodes. Subsequently, the patient is repositioned into the lateral decubitus position, and thoracoscopic ports are inserted. Approximately 20 min after ICG injection, lymphatic vessels can be visualized using a near-infrared filter on the thoracoscopic camera (4, 5). If the thoracic approaches unsuccessful or contraindicated, laparoscopic ligation of cisterna chyli offers an alternative. Various laparoscopic approaches have been described, but generally involve dissection of the right crus of the diaphragm up to the inferior vena cava (IVC), which is retracted laterally to the right. This allows exposure of the soft tissue posterior to the IVC, where the cisterna chily is typically located posteromedially. Once identified, it can be ligated and clipped (6). We report the case of a patient who underwent left pneumonectomy and subsequently developed ipsilateral chylothorax on post-operative day 16. The condition was successfully treated using ICG fluorescence for thoracic duct identification during right-sided video-assisted thoracoscopic surgery (VATS).

## Case report

**Figure 1:**
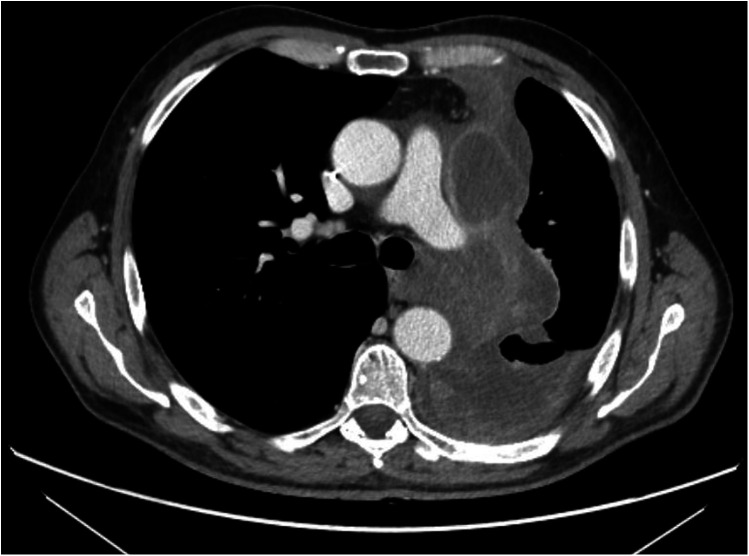
Preoperative CT-scan.

A 62-year-old male with SMARCB1-deficient mediastinal sarcoma, previously treated with neoadjuvant chemotherapy, underwent left pneumonectomy via median sternotomy and thoracotomy due to hilar infiltration (Figure 1).

**Figure 2:**
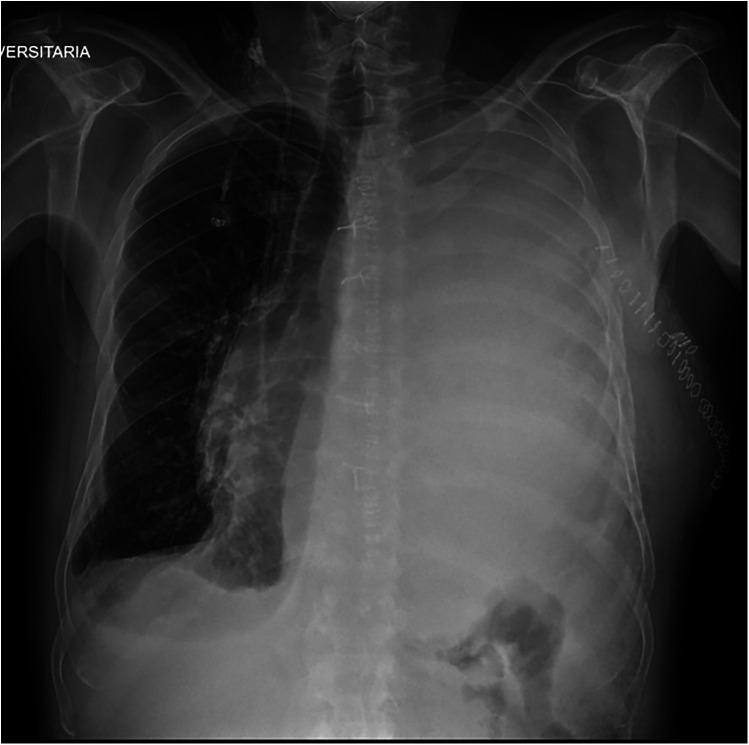
Chest x-ray showing massive left pleural effusion.

Postoperative histological examination revealed residual undifferentiated sarcoma with high malignancy. Following surgery, the patient was transferred to the intensive care unit. He resumed a normal diet on postoperative day 3, and the chest tube was removed on postoperative day 4. On postoperative day 5, he developed pericardial effusion secondary to mediastinal compression from a massive pleural effusion, which led to hemodynamic instability. This required surgical revision of the left thoracic cavity via uniportal video assisted thoracic surgery (VATS) to completely drainage of the pleural effusion. The patient was transferred back to our department on postoperative day 9. On postoperative day 11, the chest drain was removed after recording a serous output of 200 ml/day. However, the patient was not deemed ready for discharge the following day due to the need for further adjustment of his cardiac therapy. On postoperative day 16, the patient presented with dyspnea and oxygen desaturation. Chest x-ray revealed a massive pleural effusion accompanied by a contralateral mediastinal shift (Figure 2). A new chest drain was inserted, and chylothorax was diagnosed based on visual inspection as well as chemical and physical analysis of pleural fluid. Initial conservative management including fasting and parenteral nutrition, an artificial nutrition method involving intravenous administration of nutrients, were started and were continued for 21 days.

Specifically, the patient received Clinimix N14, a low-fat parenteral nutrition formulation, via central venous catheter, without resolution of the condition. Two attempts at lymphography with embolization of the lymphatic vessels were performed by the Interventional Radiologist on postoperative days 21 and 28, both unsuccessful in controlling the leak. As a result, on postoperative day 37 surgical intervention was undertaken. Thoracic duct ligation was performed using a right-sided VATS approach with the aid of ICG fluorescence for intraoperative identification of the thoracic duct. General anesthesia was administered, and orotracheal intubation was performed using a single-lumen endotracheal tube with a bronchial blocker positioned at the level of the right lower bronchus to selectively exclude the right lower lobe and facilitate the surgical procedure. For ICG fluorescence imaging, interventional radiologists injected 3 mg of ICG diluted in 5 ml of saline into the inguinal lymph nodes, which were identified using ultrasound guidance. The patient was then repositioned in left lateral decubitus for surgery. A triple-port right-sided VATS was performed using a 3D camera equipped with an ICG fluorescence filter.

**Figure 3:**
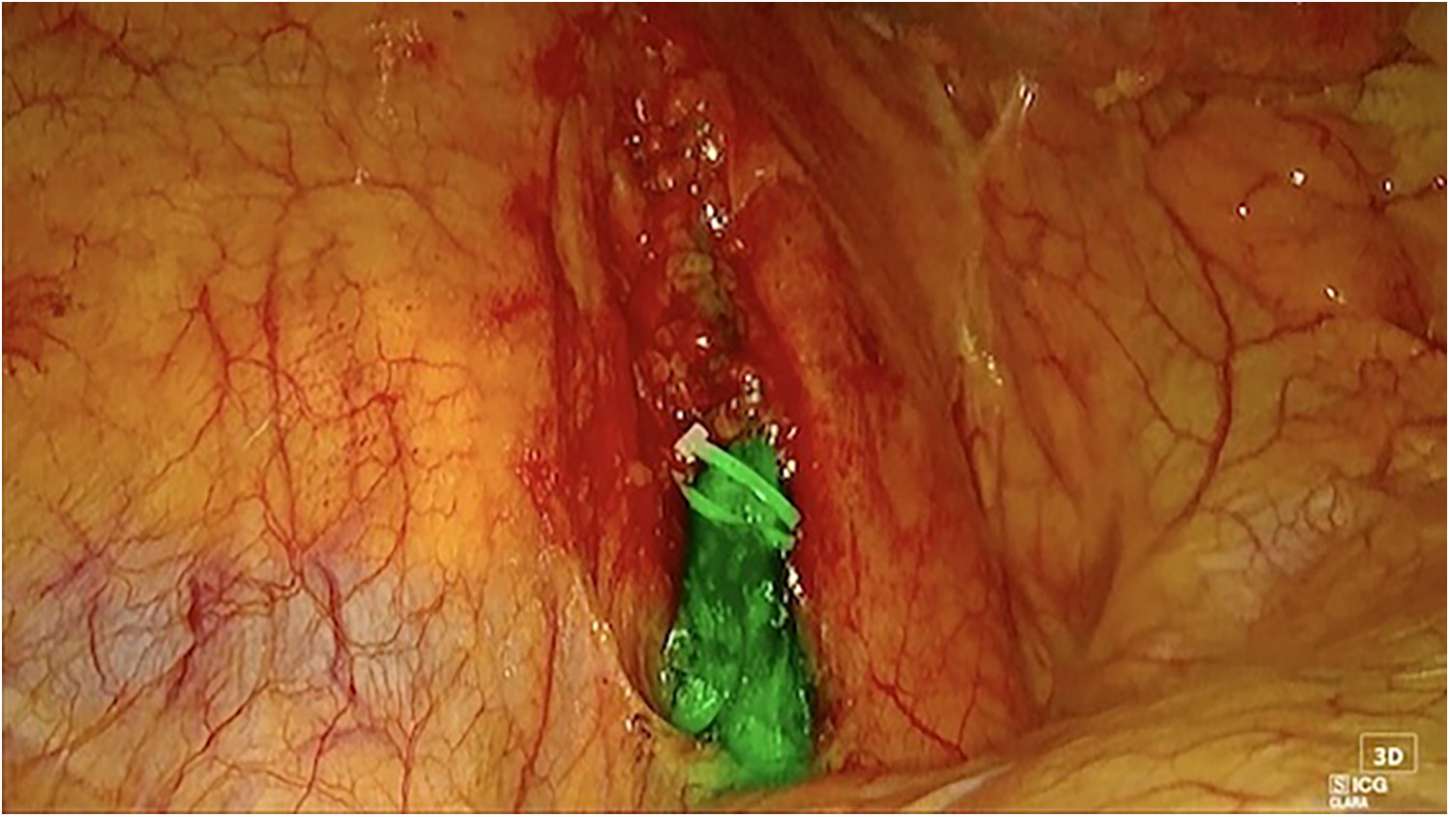
Right thoracoscopic view of the main thoracic duct. Both hem-o-lok and endo-clips are positioned for thoracic duct closure.

**Figure 4:**
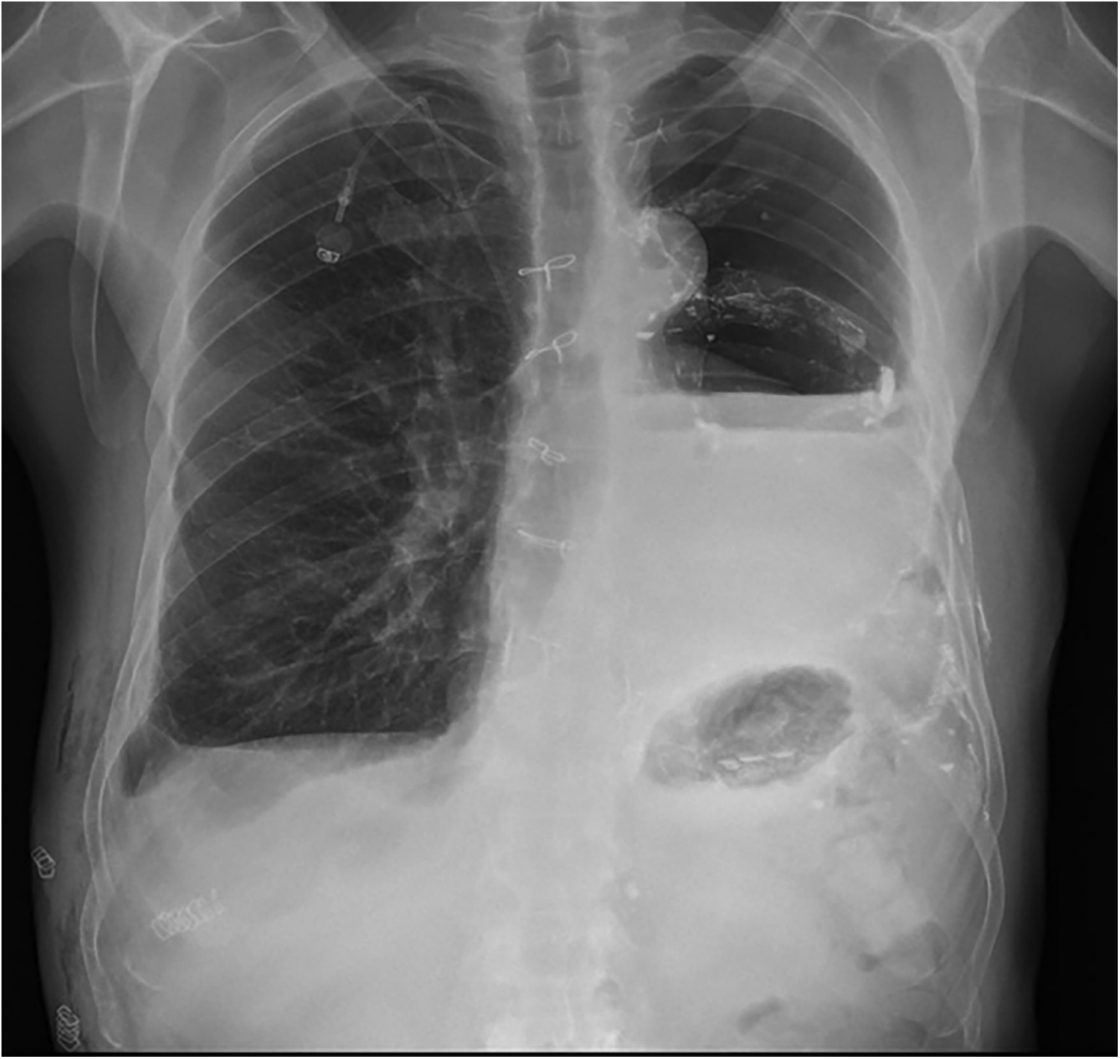
Control chest x-ray before discharge.

The thoracic duct was clearly visualized and successfully ligated using hem-o-loks and metal endoclips (Figure 3). Postoperatively, the patient resumed a full oral diet the day after surgery, with no recurrence of chylothorax. The right thoracic drain was removed on the same day as surgery, while the left thoracic drain was removed on postoperative day 2. The patient was discharged on postoperative day 40 following the initial surgery, and on postoperative day 3 after the thoracic duct ligation with a satisfactory control chest x-ray (Figure 4). The patient returned to our outpatient clinic 10 days after discharge for removal of surgical sutures. On that occasion the final histopathological results were discussed, confirming the diagnosis of SMARCB1-deficient mediastinal sarcoma. The patient was subsequently referred to the Oncology Unit for continued clinical and radiological follow-up. All clinical timepoints are summarized in Table 1.

**Table 1:** Clinical timepoints of case report.

Date	Event/action
Day 0	Surgery: left pneumonectomy
Postoperative day 1	Normal diet was initiated
Postoperative day 4	Chest tube removal
Postoperative day 5	Massive pleuro- pericardial effusion requiring surgical revision with chest tube replacement
Postoperative day 11	Chest tube was removed (after second surgery)
Postoperative day 16	Development of chylothorax with new chest tube placement
Postoperative day 21	First lymphography was performed but unsuccessful
Postoperative day 28	Second lymphography was performed but unsuccessful
Postoperative day 37	Thoracic duct ligation (through right -sided VATS) with ICG
Postoperative day 37	Right chest tube removal (after thoracic duct ligation)
Postoperative day 39	Left chest tube removal (inserted as soon as chylothorax was detected)
Postoperative day 40	Discharge

VATS, video-assisted thoracic surgery; ICG, indocyanine green.

## Discussion and conclusions

Chylothorax is a well-recognized complication following lung resections and can be a serious condition, potentially leading to malnutrition and immunosuppression (7). Conservative management typically serves as the first-line approach for treating chylothorax (8). In addition, lymphography is a valuable diagnostic and therapeutic tool; embolization of lymphatic vessels with lipiodol can occasionally resolve the condition (9). However, when both conservative and lymphographic treatments prove ineffective, thoracic duct ligation becomes necessary. Intraoperative use of ICG fluorescence has emerged as a promising technique for identifying the thoracic duct and its afferent branches during surgery. ICG is employed across various surgical specialties to evaluate anastomotic perfusion and to visualize critical anatomical structures, including lymphatic vessels and pulmonary nodules (10). Several studies have demonstrated the efficacy of ICG fluorescence in thoracic surgery to enhance visualization and facilitate accurate identification of the thoracic duct. Tappei et al. (11) described thoracoscopic thoracic duct ligation using ICG fluorescence as the only curative approach in a patient with idiopathic chylothorax. Excellent results were also reported by Benoit et al. (10), who described the resolution of chylothorax with this technique following radical neck dissection. Londero et al. (4) presented two case reports addressing the treatment of post-operative chylothorax: the first involved the identification and ligation of an afferent vessel of the thoracic duct after para-aortic lymphadenectomy, while the second described a chylothorax that occurred after a right upper bilobectomy, which was successfully treated with a thoracic duct ligation via re-VATS. All the thoracic surgical approaches mentioned above were performed using ICG fluorescence and resulted in complete clinical resolution without post-operative complications. The peculiarity of this case report lies in the successful treatment of a chylothorax following a left pneumonectomy through a right-sided VATS approach, using ICG fluorescence to close the thoracic duct. In the literature, the standard approach for thoracic duct ligation after pneumonectomy typically involves reusing the previous surgical accesses (either thoracotomy or VATS) without the aid of ICG fluorescence. Le Pimpec et al. (12) described a case series of chylothorax following lung resections, managed by direct closure of the afferent lymphatic vessel via redo thoracic surgical access. Similarly, Tokunaga et al. (13) reported a single case of chylothorax after a left pneumonectomy, which was treated by direct closure of the afferent lymphatic vessels through a redo left thoracotomy.

In our case, ICG fluorescence was of paramount importance for the precise identification of the thoracic duct on the right side, allowing for targeted ligation. Due to the patient's previous left pneumonectomy, exclusion of the entire right lung was not feasible. Therefore, we opted to isolate only the right lower lobe using a bronchial blocker. Although unconventional, this approach is innovative and underscores the adaptability required in managing complex clinical scenarios. Furthermore, this type of surgical strategy emphasizes the critical role of a multidisciplinary team- including radiologists, anesthesiologists and surgeons- in delivering optimal patient care.

This case highlights the effectiveness of ICG fluorescence in facilitating the identification and ligation of the thoracic duct in complex cases of chylothorax. It also underscores the potential for a tailored surgical approach in patients with unique anatomical challenges, such as those who have undergone pneumonectomy. In such patients, a redo-thoracotomy presents significant difficulties from both a surgical and anesthetic perspectives. Surgically, re-entering the thoracic is complicated by the presence of pleural adhesions and scarring tissue, which increase the risk of injuries and prolong operative time. From the anesthesiologist's standpoint, prolonged one-lung ventilation can predispose the patient to post-operative complications, including ARDS (Acute respiratory distress syndrome). For these reasons, our decision to pursue a contralateral surgical approach represents a safer option, aiming to minimize the duration of one -lung ventilation and reduce the associated risks. Additionally, it allowed us to operate in a pleural cavity that had not been altered by previous surgical interventions. In conclusion, ICG fluorescence can expedite the surgical procedure and significantly enhance the safety of thoracic duct ligation, particularly in cases where reducing operative time is essential. Furthermore, the management of such complex clinical scenarios highlights the critical importance of a multidisciplinary team approach including surgeons, anesthesiologists, and radiologists to ensure a tailored and optimized treatment plan for each patient.

## Data Availability

The raw data supporting the conclusions of this article will be made available by the authors, without undue reservation.
